# Increased plasma concentration of vascular endothelial growth factor in patients with atopic dermatitis and its relation to disease severity and platelet activation

**DOI:** 10.1007/s00011-012-0543-6

**Published:** 2012-08-23

**Authors:** E. Koczy-Baron, J. Jochem, A. Kasperska-Zajac

**Affiliations:** 1 Chair and Clinical Department of Internal Diseases, Allergology and Clinical Immunology, Medical University of Silesia, ul. Ceglana 35, 40-952 Katowice, Poland; 2 Department of Dermatology and Venerology in Bytom, Bytom, Poland; 3 Department of Basic Medical Sciences, Medical University of Silesia, ul. Piekarska 18, 41-902 Bytom, Poland

**Keywords:** Atopic dermatitis, Platelet-poor plasma, Vascular endothelial growth factor, Platelet activation

## Abstract

**Background:**

Overproduction of vascular endothelial growth factor (VEGF) in atopic dermatitis (AD) lesions has previously been observed. It is also known that platelet is an important source of VEGF and platelet factor 4 (PF-4), a potential marker of AD severity.

**Aim:**

To evaluate concentrations of VEGF and its soluble receptors (sVEGF-R1 and sVEGF-R2) in the plasma of AD patients and to examine its possible correlation with disease severity and plasma concentrations of PF-4, a platelet activation marker.

**Methods:**

Plasma concentrations of VEGF and its receptors and levels of PF-4 were measured by an immunoenzymatic assay in 51 AD patients and in 35 healthy non-atopic controls. The severity of the disease was evaluated using the eczema area and severity index.

**Results:**

AD patients showed significantly increased VEGF and PF-4 plasma concentrations as compared with the controls. Plasma concentrations of sVEGF-R1 and sVEGF-R2 did not differ between the groups. There were no remarkable correlations between plasma VEGF concentration and disease severity or between VEGF and PF-4 concentration.

**Conclusions:**

This study shows that plasma concentration of VEGF may be increased in patients suffering from AD. It seems that plasma VEGF concentration is not a useful marker of disease severity and, apart from platelets, other cells might also release the cytokine.

## Introduction

Atopic dermatitis (AD) is a chronic inflammatory skin disease which results from interaction of skin barrier defects, Th1/Th2 cells dysregulation, and environmental factors. Histologically, it is characterized by dilated vessels and perivascular edema leading to erythema and edema [1]. Interestingly, based on the mouse model of AD, it has been observed that angiogenesis is the major pathologic feature of the disease [2]. It has also been suggested that mast cells in AD may stimulate neoangiogenesis via the release of proangiogenic factors [3]. It is known that the key role in vascular permeability, vasodilation and angiogenesis is played by vascular endothelial growth factor (VEGF) [4]. It may also stimulate inflammatory cell recruitment, enhance antigen sensitization and appear crucial for adaptive T(H)2 inflammation [5]. There are few data available on the role of VEGF in AD. Zhang et al. [6] demonstrated increased production of VEGF in AD lesions. Zablotna et al. [7] suggested an association between the –1154 VEGF gene polymorphism and AD. Therefore, the objective of our study was to evaluate concentrations of VEGF and its soluble receptors (sVEGFR1 and VEGFR2) in plasma of patients with AD and to examine their possible correlation with disease severity. Because platelets are important sources of VEGF, we assessed the relationship between plasma concentrations of platelet factor 4 (PF-4), a platelet activation marker, and this cytokine.

## Methods

### Patients

Fifty-one patients who fulfilled the AD criteria as defined by Rajka and Langeland [8] were enrolled into the study. Their clinical and laboratory baseline characteristics are shown in Table 1. The patients were examined during the active period of the disease. Disease severity was assessed according to the eczema area and severity index (EASI) scoring system [9]. The majority of the patients (33) also suffered from persistent allergic rhinitis without any asthma symptoms. The remaining patients suffered from AD without any other atopic diseases such as asthma, rhinitis or conjunctivitis. All the patients were sensitized to house dust mite (HDM) allergens; positive skin tests to HDM (*Dermatophagoides pteronyssinus* and/or *Dermatophagoides farinae*) extracts and positive serology (specific IgE = class 2 or higher). They also showed positive skin prick tests to other inhalant and food allergens which, however, were not clinically significant.

**Table 1:** Clinical and laboratory characteristic of AD patients

Parameters	AD whole group
*N*	51
Sex (F/M)	29/22
Age (range; years)	23 (18–35)
Disease duration (range; years)	20 (4–31)
Concomitant diseases	
PAR	33
Asthma	0
Others	0
Total IgE (IU/ml) (range)	522 (153–3,350)
IgE anti-*Der p* (IU/ml) (range)	8.37 (1.2–48.2)
IgE anti-*Der f* (IU/ml) (range)	11.22 (1.1–50)
SPT positive	
House dust mites*	51 (100 %)
Animal dander	24 (47.05 %)
Moulds	6 (11.76 %)
Trees	19 (37.25 %)
Grasses	33 (64.70 %)
Weeds	14 (27.45 %)
Foods	6 (11.76 %)

Data are presented as median and range

*N* number of patients, *AD* atopic dermatitis, *PAR* persistent allergic rhinitis, *SPT* skin prick tests, *Der p* Dermatophagoides pteronyssinus, *Der f* Dermatophagoide farinae, *IgE* immunoglobulin E

* *Der p* and *Der f*

The patients were not treated with any antihistamines, topical steroids or calcineurin inhibitors for at least 1 week before enrolment into the study (only emollients were applied). They were free of any systemic steroids during the preceding 8 weeks.

The patients were compared with 35 healthy non-atopic subjects (20 males, 15 females) aged 18–38 years (median 21 years). None of the subjects had any other concomitant dermatological or medical disorders.

All the subjects submitted respective written consent and the study was approved by the University Committee of Ethics.

### Blood samples and analytical methods

Because platelets are a potential source of PF-4 and VEGF, we measured VEGF concentration in platelet-poor plasma (PPP). Blood was obtained in the morning (07:00 to 08:00, in the fasting state) after a 25-min rest at slight or no stasis from the antecubital vein into CTAD tubes containing four anticoagulants—sodium citrate, theophylline, adenosine and dypiridamole (Vacutainers^®^, Becton–Dickinson) to obtain maximal stabilization of platelets, then placed into an ice/water bath. The tubes were then centrifuged at 3,000×*g* for 15 min at 4 °C. Following the first centrifugal cycle three-quarters of the top plasma was removed with a plastic transfer pipet. This plasma was centrifuged again at 3,000×*g* for 15 min to remove the residual platelets. The plasma obtained was stored at −70 °C until assayed for VEGF and PF-4.

Measurement of PF-4 concentration in PPP was performed to assess the degree of platelet activation in vivo.

sVEGF-R1 and sVEGF-R2 concentrations were performed in the plasma collected using EDTA as an anticoagulant.

### VEGF analysis

VEGF plasma concentrations were determined using the Quantikine Human VEGF enzyme-linked immunosorbent assay (ELISA) (R&D Systems Inc., Minneapolis, MN, USA), to recognize the soluble isoforms (VEGF121 and VEGF165). The detection limits were 9.0 pg/ml. Values <9 pg/ml were equalized to zero.

### sVEGF-R1 and sVEGF-R2 analyses

The receptor plasma concentrations were assayed by specific commercially available ELISA assay kits (Quantikine; R&D Systems Inc.) in accordance with the manufacturer’s instructions. The sensitivity of the assay for VEGF-R1 and sVEGFR-2 was 3.0 and 5 pg/ml, respectively.

### PF-4 analysis

The PF-4 concentration was measured in the PPP by ELISA using commercial Asserachrom^®^ (Diagnostica Stago, France). The detection limit was 0.25 IU/ml.

### Skin prick tests

Allergic status was evaluated using a panel of common inhalants and the main food allergens (Allergopharma, Reinbeck, Germany). The skin wheal-flare reaction was read after 15 min and considered positive if the wheal diameter was at least 3 mm larger than one formed by the control substance.

### Other laboratory investigations

The serum levels of total immunoglobulin E (IgE) and specific IgE to *D. farinae* and *D. pteronyssinus* were measured by ELISA using a commercial kit (Allergopharma) according to the manufacturer’s instructions. The blood platelet and eosinophil counts were determined using an automatic hematology analyzer.

### Statistical analysis

Data are presented as median and ranges. All the statistical evaluations were performed by Mann–Whitney *U* test. The correlations between parameters were measured with Spearman rank test. The results were considered significant when *P* < 0.05.

### Results

**Fig. 1:**
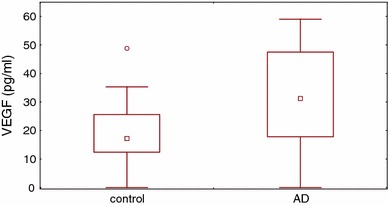
Plasma VEGF concentration was significantly higher in AD patients as compared with controls (*P* = 0.0007)

**Fig. 2:**
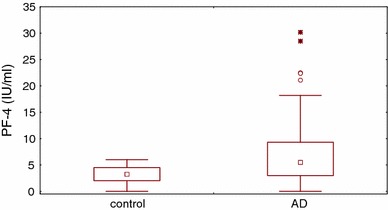
Plasma PF-4 concentration was significantly higher in AD patients as compared with controls (*P* = 0.0005)

VEGF plasma concentration was significantly higher in AD patients than in the healthy controls (31.2 and 17.2 pg/ml, respectively; *P* = 0.0007; Fig. 1). Plasma concentrations of VEGF-R1 and VEGF-R2 did not differ significantly between AD patients and healthy subjects (36.2 vs 35.8 and 8,145 vs 7,470 pg/ml, respectively). There were no significant differences in plasma concentrations of VEGF, sVEGF-R1 and sVEGF-R2 between AD patients with and without persistent allergic rhinitis. Plasma concentration of PF-4 was significantly increased in AD patients as compared with the controls (5.5 and 3.2 IU/ml, respectively, *P* = 0.0005; Fig. 2). No significant correlation was found between VEGF and PF-4 (*r* = 0.26, *P* = 0.6). There were no correlations between VEGF and VEGF-R1 or VEGF-R2 or between VEGF-R1 and VEGF-R2. In addition, no significant correlation was found between VEGF concentration and the counts of platelets and eosinophils (data not shown). Neither did we observe any significant correlation between plasma VEGF and serum concentration of total IgE and specific IgE anti-HDM (data not shown).

Plasma VEGF concentration did not correlate significantly with EASI.

### Discussion

These findings demonstrate for the first time that patients with AD may show significantly increased plasma concentration of VEGF. Because platelets are important sources of VEGF in the circulation [10, 11], we performed the analysis in PPP. At present, the major sources and the role of increased plasma concentration of VEGF in AD patients are unknown and remain speculative.

Different cells involved in the pathogenesis of AD are able to synthesize and release VEGF.

Overproduction of VEGF in AD lesional keratinocytes has been demonstrated. The amount of VEGF produced in the lesions of AD was approximately 25 times higher than in normal stratum corneum; however, the mechanism is unclear [6]. It is therefore possible that keratinocytes in AD might release greater amounts of VEGF, which in turn could contribute to the subsequent increase in plasma concentration. Another important storage site is formed by the platelets, which release VEGF upon activation in vivo. It has been reported that platelet activation measured by plasma concentrations of PF-4 and beta-thromboglobulin is increased in AD patients [12–15], but not in other manifestations of atopic diathesis [16, 17]. In addition, it has been suggested that chemokines are markers of AD severity [13]. Our study indicated no significant correlation between plasma concentrations of VEGF and PF-4, suggesting that platelets are not likely to be the sole source of VEGF in AD. It has been demonstrated that activated eosinophils may appear to be an important source of the vascular permeability factor, which may contribute to tissue edema at the sites of allergic inflammation [18]. In addition, sVEGFR1 is expressed by eosinophil whose activation with VEGF stimulates directed migration and activation of eosinophil. Thus, VEGF may play an important role in the modulation of eosinophilic inflammation [19]. In our study, there was no significant correlation between plasma concentration of VEGF and eosinophil counts, suggesting that eosinophils could not be the sole source of VEGF in AD.

Other possible sources of VEGF include mast cells. Mast cells can secrete VEGF and such secretion is enhanced via upregulation of IgE receptor on mast cells [20]. Interestingly, it has been suggested that transfer of IgE from the circulating blood to extravascular tissue via endothelial cells may depend on the concentration of VEGF secreted from mast cells [21]. We did not observe any significant correlation between plasma VEGF and serum concentration of total IgE and specific IgE anti-HDM. Furthermore, other cells involved in immune inflammatory processes in AD can release VEGF. Different cell sources probably contribute to the subsequent increase in plasma concentration of VEGF.

Our results show that AD severity according EASI does not correlate with plasma VEGF concentration. This might suggest the lack of an important link between the degree of skin inflammation and VEGF release. VEGF plasma concentration may not be a useful marker of disease severity.

It has been suggested that VEGF may play a role in the pathogenesis of AD and may regulate the development of AD lesions, acting possibly in the persisting erythema and edema by prolonged capillary dilatation and hyperpermeability [6]. Apart from AD, increased expression of VEGF has been observed in patients suffering from other inflammatory skin diseases associated with enhanced vascularity and vascular hyperpermeability, including bullous pemphigoid, dermatitis herpetiformis and erythema multiforme [22]. Overexpression of VEGF and its receptors has been observed in delayed hypersensitivity skin reactions [23].

The significance of the increased concentration of circulating VEGF in AD is unclear. Whether circulating VEGF contributes directly or indirectly to AD pathogenesis or is merely a secondary phenomenon needs to be determined. Because VEGF is a multifunctional cytokine secreted by a variety of cells and is overexpressed in AD, it leads to the hypothesis that circulating VEGF may be involved in AD and provides a link between vascular permeability and leukocyte recruitment as well as activation at sites of the inflammation.

There was no correlation between plasma concentrations of VEGF and its soluble receptors (sVEGF-R1 and sVEGF-R2) in AD patients.

Considering that sVEGF-R1 is a negative regulator of VEGF availability (by sequestrating the ligand and by forming inactive heterodimers with membrane-bound VEGF receptors), one could expect some changes in plasma sVEGF-R1 concentration shown by AD patients. No increased plasma sVEGF-R1 concentration observed in this study may suggest a paradoxical response of sVEGF-R1, promoting the VEGF performance. Such a phenomenon could hypothetically account for the disturbance of mechanisms responsible for VEGF activity in the VEGF/sVEGF-R1 system in AD patients. Our results are not sufficient, however, to draw any ultimate conclusions, particularly because data illustrating a correlation between the concentration of VEGF and its receptors are scarce, if not divergent.

On the other hand, the function of sVEGF-R2 is less recognized, while in vitro it appears as a weak antagonist of VEGF [24].

## Conclusions

The study appears as the first reported evidence of an increased concentration of VEGF in PPP in atopic dermatitis; however, its role remains uncertain and further investigations should be undertaken for better recognition of its function. The involvement of VEGF in the inflammatory reaction of AD might be supported by evidence of a variety of biological effects exerted by VEGF on cells and processes that play a major role in AD. Different cells sources probably contribute to the subsequent increase in plasma concentration of VEGF. The major source of circulating VEGF in AD is still unknown. It seems that plasma VEGF concentration is not a useful marker of disease severity and, apart from platelets, other cells might also release the cytokine.

## References

[1] Leung DY, Bieber T (2003). Atopic dermatitis. Lancet.

[2] Agha-Majzoub R, Becker RP, Schraufnagel DE, Chan LS (2005). Angiogenesis: the major abnormality of the keratin-14 IL-4 transgenic mouse model of atopic dermatitis. Microcirculation.

[3] Groneberg DA, Bester C, Grutzkau A, Serowka F, Fischer A, Henz BM, Welker P (2005). Mast cells and vasculature in atopic dermatitis–potential stimulus of neoangiogenesis. Allergy.

[4] Takahashi H, Shibuya M (2005). The vascular endothelial growth factor (VEGF)/VEGF receptor system and its role under physiological and pathological conditions. Clin Sci (Lond).

[5] Lee CG, Link H, Baluk P, Homer RJ, Chapoval S, Bhandari V, Kang MJ, Cohn L, Kim YK, McDonald DM, Elias JA (2004). Vascular endothelial growth factor (VEGF) induces remodeling and enhances TH2-mediated sensitization and inflammation in the lung. Nat Med.

[6] Zhang Y, Matsuo H, Morita E (2006). Increased production of vascular endothelial growth factor in the lesions of atopic dermatitis. Arch Dermatol Res.

[7] Zablotna M, Sobjanek M, Glen J, Niedoszytko M, Wilkowska A, Roszkiewicz J, Nedoszytko B (2010). Association between the −1154 G/A promoter polymorphism of the vascular endothelial growth factor gene and atopic dermatitis. J Eur Acad Dermatol Venereol.

[8] Hanifin J, Rajka G (1980). Diagnostic features of atopic dermatitis. Acta Derm Venereol (Stockh).

[9] Hanifin JM, Thurston M, Omoto M, Cherill R, Tofte SJ, Graeber M (2001). The eczema area and severity index (EASI): assessment of reliability in atopic dermatitis. EASI Evaluator Group.. Exp Dermatol.

[10] Jelkmann W (2001). Pitfalls in the measurement of circulating vascular endothelial growth factor. Clin Chem.

[11] Nielsen HJ, Werther K, Mynster T, Brunner N (1999). Soluble vascular endothelial growth factor in various blood components. Transfusion.

[12] Kasperska-Zajac A, Nowakowski M, Rogala B (2004). Enhanced platelet activation in patients with atopic eczema/dermatitis syndrome. Inflammation.

[13] Tamagawa-Mineoka R, Katoh N, Ueda E, Masuda K, Kishimoto S (2008). Elevated platelet activation in patients with atopic dermatitis and psoriasis: increased plasma levels of beta-thromboglobulin and platelet factor 4. Allergol Int..

[14] Kasperska-Zajac A (2010). Recovery of platelet factor 4 (PF-4) and beta-thromboglobulin (beta-TG) plasma concentrations during remission in patients suffering from atopic dermatitis. Platelets.

[15] Kasperska-Zajac A, Brzoza Z, Rogala B (2008). Platelet function in cutaneous diseases. Platelets.

[16] Kasperska-Zajac A, Rogala B (2005). Markers of platelet activation in plasma of patients suffering from persistent allergic rhinitis with or without asthma symptoms. Clin Exp Allergy.

[17] Kasperska-Zajac A, Rogala B (2003). Platelet activity measured by plasma levels of beta-thromboglobulin and platelet factor 4 in seasonal allergic rhinitis during natural pollen exposure. Inflamm Res.

[18] Horiuchi T, Weller PF (1997). Expression of vascular endothelial growth factor by human eosinophils: upregulation by granulocyte macrophage colony-stimulating factor and interleukin-5. Am J Respir Cell Mol Biol.

[19] Feistritzer C, Kaneider NC, Sturn DH, Mosheimer BA, Kahler CM, Wiedermann CJ (2004). Expression and function of the vascular endothelial growth factor receptor FLT-1 in human eosinophils. Am J Respir Cell Mol Biol.

[20] Boesiger J, Tsai M, Maurer M, Yamaguchi M, Brown LF, Claffey KP, Dvorak HF, Galli SJ (1998). Mast cells can secrete vascular permeability factor/vascular endothelial cell growth factor and exhibit enhanced release after immunoglobulin E-dependent upregulation of fc epsilon receptor I expression. J Exp Med.

[21] Nakasone T, Hanashiro K, Nakamura M, Sunakawa H, Kosugi T (2002). Mast cell-derived VEGF enhances the passage of IgE FE-3 through the rat aortic endothelial cell monolayer. Int Arch Allergy Immunol.

[22] Brown LF, Harrist TJ, Yeo KT, Stahle-Backdahl M, Jackman RW, Berse B, Tognazzi K, Dvorak HF, Detmar M (1995). Increased expression of vascular permeability factor (vascular endothelial growth factor) in bullous pemphigoid, dermatitis herpetiformis, and erythema multiforme. J Invest Dermatol.

[23] Brown LF, Olbricht SM, Berse B, Jackman RW, Matsueda G, Tognazzi KA, Manseau EJ, Dvorak HF, Van de Water L (1995). Overexpression of vascular permeability factor (VPF/VEGF) and its endothelial cell receptors in delayed hypersensitivity skin reactions. J Immunol.

[24] Roeckl W, Hecht D, Sztajer H, Waltenberger J, Yayon A, Weich HA (1998). Differential binding characteristics and cellular inhibition by soluble VEGF receptors 1 and 2. Exp Cell Res.

